# Evaluation of Orthopaedics and Traumatology Patients Referred From a Tertiary Hospital

**DOI:** 10.7759/cureus.47904

**Published:** 2023-10-29

**Authors:** Hakan Yolaçan, Serkan Guler

**Affiliations:** 1 Orthopaedics and Traumatology, Aksaray University Training and Research Hospital, Aksaray, TUR

**Keywords:** tertiary hospital, emergency service, transfer, interhospital transfer, orthopaedics and traumatology

## Abstract

Background

This study aims to determine the rate of interhospital transfer, transfer diagnoses, where they were referred, and the reasons for the transfer of patients who presented to the emergency department and requested orthopaedic and traumatology consultations and to evaluate measures that may be effective in reducing the number of referrals for a more effective health service provision as a result of this information.

Material and methods

In this descriptive study, 59 patients were transferred to the emergency department of our hospital between January 1, 2019, and January 1, 2022, for whom orthopaedic and traumatology consultations were requested and for whom the orthopaedic physician requested transfer (training and research hospitals, university hospitals and private hospitals), and they were retrospectively evaluated.

Results

The ages of the study participants ranged from 1 to 91 years, with a mean age of 39.8 ± 20.9 years. Therefore, the majority of referred patients were male and Turkish citizens aged 18-65 years; there were no forensic cases; they were evaluated in the yellow area as a result of triage; and most of the transfers were from training and research hospitals and university hospitals, which are tertiary health institutions. On categorising patients based on their transfer diagnoses, it was found that patients with subtotal amputation of the finger were the most common among the referred patients.

Conclusion

To reduce the number of referrals, it is recommended to increase the standards in assistant training, especially in pelvis, acetabulum, and hand surgeries. It is also recommended to optimise material supply and skilled labour distribution.

## Introduction

In our country’s current health system, patients are usually transferred to the nearest hospital via the 112 emergency service. If the necessary medical intervention cannot be performed in the hospital requested (because of a lack of medical devices, intensive care conditions, bed occupancy, the need for a specific surgical intervention, etc.), the patient is transferred to another centre via the 112 emergency service.

As specialised branch hospitals and advanced medical equipment have been developed [1], the demand for hospital transfers has increased. Reportedly, 10%-35% of all transfers within the ambit of emergency health services are interhospital transfers [2]. According to Kindermann et al., 1.5% of patients admitted to the emergency department are referred to a different hospital. Moreover, it is crucial to evaluate the patient quickly and in a multidisciplinary manner, considering the hospital’s physical resources and physicians’ experience [3].

Our study aims to determine the rate of interhospital transfer, transfer diagnoses, where they were referred, and the reasons for the transfer of patients who presented to the emergency department and requested orthopaedic and traumatology consultations and to evaluate measures that may be effective in reducing the number of referrals for more effective health service provision because of this information.

Our study hypothesises that the majority of referred patients have pelvic and acetabulum fractures requiring advanced specialist physicians and teams and hand surgery cases requiring a subspecialty physician.

## Materials and methods

This study was approved by the Ethics Committee of Aksaray University Training and Research Hospital in 2022, with decision 11-03. In our descriptive study, 59 patients who were transferred to the emergency department of our hospital between January 1, 2019, and January 1, 2022, for whom orthopaedic and traumatology consultations were requested and whose transfer request was made by the orthopaedic surgeon, were evaluated retrospectively.

The population of the city where the research was conducted is approximately 450,000. Although the number of patients admitted to the emergency department where the study was conducted varies according to the season, the number of patients examined annually is approximately 300,000, and the number of patients for whom annual orthopaedic and traumatology consultations were requested is around 3,000.

The study included all age-group patients who were referred by the orthopaedic and traumatology departments. The study excluded patients who did not have a reason for transfer, a transfer diagnosis, or an explanation for their transfer. In addition to demographic data, such as age, gender, and nationality, triage code, hospital information as to where the patient is to be transferred; the reason for transfer, transfer diagnosis, and forensic case status were recorded for all patients included in the study.

Statistical Analysis

The distribution based on transfer diagnoses was done manually. For statistical analysis, the Statistical Package for the Social Sciences (SPSS) (version 26.0, IBM SPSS Statistics for Windows, Armonk, NY) was used. For categorical variables, descriptive statistics were expressed as numbers and percentages. The mean, standard deviation, and minimum and maximum values were used to represent numerical variables.

## Results

A transfer request was created via the 112 emergency service for 59 of the 8,793 patients evaluated in our hospital’s emergency department between January 1, 2019, and January 1, 2022, who were requested for orthopaedic and traumatology consultations. The patient’s ages in the study ranged from 1 to 91 years, with a mean age of 39.8 ± 20.9 years. Table 1 contains descriptive data from the study, such as gender, age distribution, nationality, forensic case status, triage code, and the transferred hospital information. Therefore, most referred patients were male and Turkish citizens aged 18-65 years; there were no forensic cases; they were evaluated in the yellow area as a result of triage; and most of the transfers were to training and research and university hospitals, which are tertiary health institutions (Table 1).

**Table 1:** Distribution of descriptive data.

Descriptive Data	Number	Percentage
Gender		
Female	9	15.3
Male	50	84.7
Age		
<18 years	9	15.3
18–65 years	43	72.8
>65 years	7	11.8
Nationality		
Turkish Republic citizen	57	96.6
Non-Turkish Republic citizen	2	3.4
Forensic case		
Yes	26	44.1
No	33	55.9
Triage code		
Green	0	0
Yellow	52	88.1
Red	7	11.8
Transferred hospital information		
Training and research hospitals	13	22
University hospitals	32	54.2
Private hospitals	14	23.7

When the patients in the study were grouped based on their transfer diagnoses, it was discovered that the patients with subtotal amputation of the finger were the most common among the referred patients (Table 2).

**Table 2:** Distribution of diagnoses among transferred patients.

Diagnosis	Number	Percentage
Subtotal amputation of the finger	14	23.7
Pelvis–acetabulum fracture	13	22
Femur fracture	11	18.6
Total finger amputation	4	6.7
Hand flexor tendon injury	4	6.7
Ulnar artery injury	3	5
Tibia fracture	3	5
Elbow fractured dislocation	2	3.4
Hip dislocation	2	3.4
Humerus fracture	2	3.4
Degloving injury of the lower extremity	1	1.7
TOTAL	59	100

Transfer requests for patients were made in our study because of the need for the most subspecialty physicians (Table 3).

**Table 3:** Transfer reason-based distribution.

Reason for transfer	Number	Percentage
Need for a subspecialty physician	36	61
Need for medical equipment	8	13.5
Need for intensive care	7	11.8
Amputation–replantation	4	6.7
Patient request	4	6.7
TOTAL	59	100

## Discussion

The majority of the patients referred in our study were males and Turkish citizens aged 18-65 years; there were no forensic cases; they were evaluated in the yellow area as a result of triage of the emergency department; and the majority of the transfers were to training and research hospitals and university hospitals, which are tertiary healthcare institutions. Furthermore, it was demonstrated that the most common transfer diagnosis was the subtotal amputation of the finger, and the most common transfer request was the need for a subspecialty physician.

When the patients in our study were evaluated based on gender, it was found that males were five times more common than females. Guler et al. reported the proportion of males among referred patients was 55.8% [4]. Additionally, Gray et al. revealed that the rate of males was twice as high as that of females [5]. Lovell et al. also reported that 60.5% of referred patients were males [6]. Accordingly, the number of men was found to be higher in our study.

According to our findings, patients in the yellow area according to the triage result were responsible for 88% of transfers, and patients in the red area according to the triage result were responsible for 12%. The patient's triage code is an important factor in the occurrence of undesirable results in interhospital transfers [7]. Nacht et al. observed that 69% of the cases were red areas, and 14% of them were yellow areas [8]. Moreover, the transfer of patients with yellow triage code was at a higher rate in our study than in the literature. The most important reason for this situation was the inadequate and inappropriate distribution of crew and equipment, which was thought to increase the cost unnecessarily.

Patients frequently receive referrals to specialised hospitals that can offer more focused services, according to a study by Mascia et al. [9]. In terms of the hospitals to which they were referred, our study found that 54.2% were referred to university hospitals, 23.7% to private hospitals, and 22% to training and research hospitals. This corroborates the findings of Lomi et al. that two-thirds of patients were referred to a tertiary hospital [10]. Another similar study by Assereh et al. found that 67% of patients were referred to a tertiary hospital [11].

According to our findings, transfer requests were made primarily due to the need for subspecialty physicians. According to Gray et al., the most common reason for transfers was the need for a specialist [5]. Another study by Iwashyna revealed that the most common reason for transfers was a lack of medical equipment [12]. It is believed that the current disparities in results are because of the fact that the classifications of the reasons for transfer between countries may differ and that the meanings of certain criteria may be intertwined.

In our country, a core training programme is used to train assistant physicians who specialise in orthopaedics and traumatology. According to this programme, resident physicians receive appropriate and sufficient training based on a variety of cases, but it is expected that resident physicians will develop more competence, knowledge, and experience by making appointments in terms of specific surgical applications.

Our research is the first in our country to look into the descriptive characteristics and causality of patients referred to orthopaedics and traumatology. Our findings will aid in the more appropriate distribution of specialised physicians and advanced medical devices.

The limitations of our study are that it was retrospective, patients were not followed up after the transfer, and some diagnoses were general, thereby preventing more specific diagnostic analyses.

## Conclusions

The most important result of our study is the existence of preventable and reduceable referral availability. Under the leadership of orthopedics and traumatology professional organizations, it is recommended to increase the standards in resident training, especially in pelvis and acetabulum surgery and hand surgery. Additionally, it is recommended to optimize the supply of materials and the distribution of skilled labor. In this way, it is thought that the number of referrals requested can be reduced.
